# Continuous distraction osteogenesis device with MAAC controller for mandibular reconstruction applications

**DOI:** 10.1186/s12938-019-0655-0

**Published:** 2019-04-08

**Authors:** Shahrokh Hatefi, Milad Etemadi Sh, Yimesker Yihun, Roozbeh Mansouri, Alireza Akhlaghi

**Affiliations:** 1 Department of Mechatronics Engineering, Nelson Mandela University, Port Elizabeth, South Africa; 2 Department of Oral and Maxillofacial Surgery, Isfahan University of Medical Sciences, Isfahan, Iran; 3 Department of Mechanical Engineering, Wichita State University, Wichita, USA; 4 Center for Advanced Engineering Research, Najaf Abad Branch, Islamic Azad University, Isfahan, Iran; 5 Isfahan University of Medical Sciences, Isfahan, Iran

**Keywords:** Automatic continuous distractor, Distraction osteogenesis, Medical devices

## Abstract

**Background:**

Distraction osteogenesis (DO) is a novel technique widely used in human body reconstruction. DO has got a significant role in maxillofacial reconstruction applications (MRA); through this method, bone defects and skeletal deformities in various cranio-maxillofacial areas could be reconstructed with superior results in comparison to conventional methods. Recent studies revealed in a DO solution, using an automatic continuous distractor could significantly improve the results while decreasing the existing issues. This study is aimed at designing and developing a novel automatic continuous distraction osteogenesis (ACDO) device to be used in the MRA.

**Methods:**

The design is comprised of a lead screw translation mechanism and a stepper motor, placed outside of the mouth to generate the desired continuous linear force. This externally generated and controlled distraction force (DF) is transferred into the moving bone segment via a flexible miniature transition system. The system is also equipped with an extra-oral ACDO controller, to generate an accurate, reliable, and stable continuous DF.

**Results:**

Simulation and experimental results have justified the controller outputs and the desired accuracy of the device. Experiments have been conducted on a sheep jaw bone and results have showed that the developed device could offer a continuous DF of 38 N with distraction accuracy of 7.6 nm on the bone segment, while reducing the distraction time span.

**Conclusion:**

Continuous DF with high resolution positioning control, along with the smaller size of the distractor placed in the oral cavity will help in improving the result of the reconstruction operation and leading to a successful DO procedure in a shorter time period. The developed ACDO device has less than 1% positioning error while generating sufficient DF. These features make this device a suitable distractor for an enhanced DO treatment in MRA.

## Background

In maxillofacial reconstruction applications (MRA) different techniques have been used; autologous bone graft, allograft implantation, osteoconduction, osteoinduction, osteoprogenitor cells, and distraction osteogenesis (DO) [1–3]. In 1989, Illizarov developed the DO technique and introduced a novel limb lengthening method. Subsequently, in 1992, MacCarthy reported the first clinical case of a DO procedure on mandible [4–7]. Since then, DO has been widely used as a treatment method to generate the bone, and to fill the skeletal defects, or to correct congenital growth retardation of the bone tissue [5, 8, 9]. In MRA, DO method is a new solution to the tissue lengthening and it is getting a higher clinical attention as a technique without the need for bone graft. The main advantage of this technique is that the bone generation occurs along with the adaption of the surrounding soft tissues, moreover, a more predictable treatment outcome could be obtained [8–13]. The method starts with the bone osteotomy and the installation of the device, after the latency period, activation phase begins and gradually callus goes through the distraction force (DF). The generated gap made by distracted callus, transforms into a mature tissue called consolidation phase, and then the device is removed [14, 15]. The external fixation distractor was developed by Illizarov in 1987 [4, 16]. The major problems of extra-oral type are scar formation, infection, and nerve injuries; such issues have leaded research groups to focus on developing intra-oral devices. Research has been done and different intra-oral distractors have been developed and used [10, 17–23]. In both internal and external devices, however, the actuation is relied upon manual length adjustment with a potential error in the procedure, and low accuracy and reliability; the distractor is activated one or two times daily with a distraction rate (DR) between 0.25 to 1 mm per day [15, 24–26]. In addition, the long treatment period induces physical and psychological discomfort to the patient [5, 27]. Illizarov used a quasi-continuous method and revealed by increasing the rhythm of distraction, at a higher DR, superior results in a more rapid course of osteogenesis could be obtained [14, 16, 28, 29].

Recent studies have shown using continuous DO could significantly increase the DR and expedite the bone healing process with a higher osteogenesis quality [7, 25, 28–35]. The key elements of the automatic continuous distraction osteogenesis (ACDO) treatment are the rate and the rhythm of the distraction, the distraction vector (DV), and the output DF generated by the distractor [24, 26, 36]. Research has been done on increasing the rate and the rhythm of the process [32], reducing the activation phase duration [37], advancing the distractor’s safety [24, 28], and improving the distraction accuracy and the DV on the unilateral models [29, 38, 39]. Various movement mechanisms and actuators have been used in the design and development of ACDO devices, including; motor-based, electromechanical system [5, 12, 25, 35, 40–43], hydraulic valve [29, 44, 45], spring-mediated system [46–49], shape memory alloy [48], load cell [50], and piezoelectric motor [24]. Existing ACDO devices could successfully distract the bone with the DR up to 3 mm per day [7, 32]. Recently, research groups are focusing on improving the distraction accuracy to enable a higher DR in a DO procedure [5, 32]. In a recent animal study on minipig mandible, by increasing the distraction accuracy, the DR up to 4.5 mm per day is successfully achieved [7, 32, 34]; as a result, by decreasing the total time in a DO protocol, the risks of complications during the treatment could be reduced [44]. The tendency is also to miniaturize the distractor for submucosal or subcutaneous application especially in unfavorable anatomical regions [51]. Furthermore, reducing the size of intra-oral part of the distractor may reduce the chance of occurring tissue injuries, infections, and bone fracture [24, 27, 44]. Although developed ACDO devices have shown promising results compared to conventional manual methods, they are still limited to be used in human clinical applications. In general, further study and improvements are required, specially to maximize distraction accuracy, DR, reliability, and safety, and to minimize control complexity and size [5, 24, 35]. The hypothesis of this research is that by increasing the distraction accuracy and providing a smoother DF at a higher DR, superior results in a shorter distraction period could be achieved. In this study, a new ACDO device is designed and developed based on a lead screw and stepper motor combination to improve the distraction accuracy, the DR, and the activation phase. A novel automatic controlling method, MAAC controller [52, 53], is implemented to generate an accurate, reliable, and stable continues DF. In addition, for the intra-oral part of the device, a miniature distraction mechanism is designed and developed. A set of bench tests and simulation results are presented to validate the feasibility of the design, to assess the performance of the ex vivo model, and to identify the key engineering challenges to be addressed in further product development for animal studies and clinical applications.

## Methods

To transfer the externally generated DF to the moving bone segment (BS) on the callus, the ACDO device consists of a miniature lead screw translation mechanism (TM), a micro controller, and a flexible shielded spring-wire transition system (TS). The details of these components are discussed in the following sub-sections.

### The lead screw translation mechanism

**Fig. 1:**
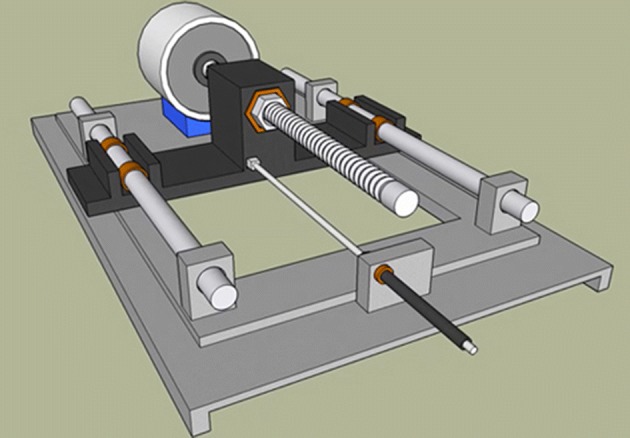
The 3D model of the designed TM

**Fig. 2:**
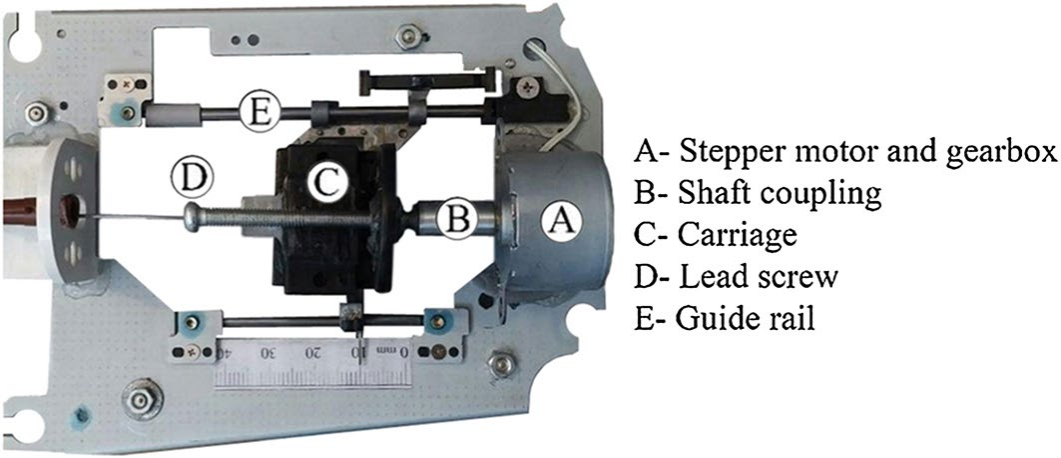
The lead screw translation mechanism

The mechatronic part of the developed ACDO device receives the movement commands from the controller and generates the linear DF. Based on the design of the mechanism a 3D model is sketched to show the system’s functionality (Fig. 1). This unit consists of a Kiatronics 28BYJ-48 mini stepper motor and gearbox (code: 70289) with specifications shown in Table 1. The gearbox is connected to a 4-mm solid shaft coupling to transmit the generated power from the stepper motor’s shaft to the screw thread. To generate the translation motion in a linear axis, a leadscrew of 4 mm diameter, right hand internal- and external-screw thread with 1-mm lead, 1-mm pitch, and length of 50 mm, and a carriage are used, as shown in Fig. 2. This configuration changes the rotation motion into a translation based on the specified DO parameters and generates a linear force. The controller could drive the stepper motor in three working states with varied linear and angular step movement, as shown in Table 2.

**Table 1:** 28BYJ-48 stepper motor specifications

	28BYJ-48 stepper motor		
Rated voltage	5 V DC	Shaft stride angle	0.088°
Number of phases	4	Rotor stride angle	5.625°
Current	4*10^−2^ A	In-traction torque	> 34.3*10^−3^ N m
DC resistance	54 Ω	Friction torque	0.12 N m
Phase inductance	3*10^−3^ H	Pull in torque	0.06 N m
Frequency	100 Hz	Insulated resistance	> 10 MΩ (500 V)
Speed variation ratio	1/64	Noise	< 35 dB

**Table 2:** The positioning accuracy of the system

Drive mode	Rotor stride angle (°)	Shaft stride angle (°)	Positioning accuracy (nm)	Carriage movement (nm/step)
Full-step (1/1)	5.625	0.088	244.14	244.14
Half-step (1/2)	2.8125	0.044	122.07	122.07
Micro-step (1/32)	0.176	0.00275	7.63	7.63

**Fig. 3:**
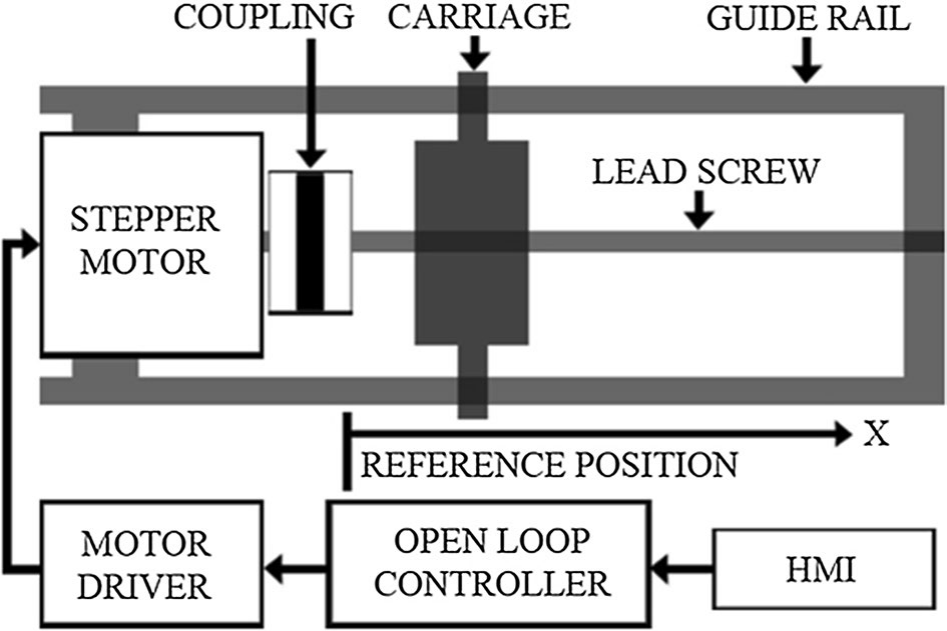
The schematic model of the mechatronic part [54]

The positioning accuracy of the system can be calculated by considering parameters containing stepper motor’s stride angle, mechanical gearbox ratio (1/64), and the TM movement accuracy (1 mm/revolution); the movement accuracy of the developed ACDO device is 244.14 nm/step in full-step drive mode, 122.07 nm/step in half-step drive mode, and 7.63 nm/step in micro-step drive mode. Micro-step mode, the most accurate driving method, is selected for running the system, which means for 1 mm of the distraction length (DL), the motor is driven by the controller for 131072 steps to complete the travel. The converted linear DF is transferred to the installed mechanical distractor on the callus in order to move the BS in a desired DV with predetermined factors. Figure 3 shows the schematic model of the mechatronic part of the device.

### Controller

**Fig. 4:**
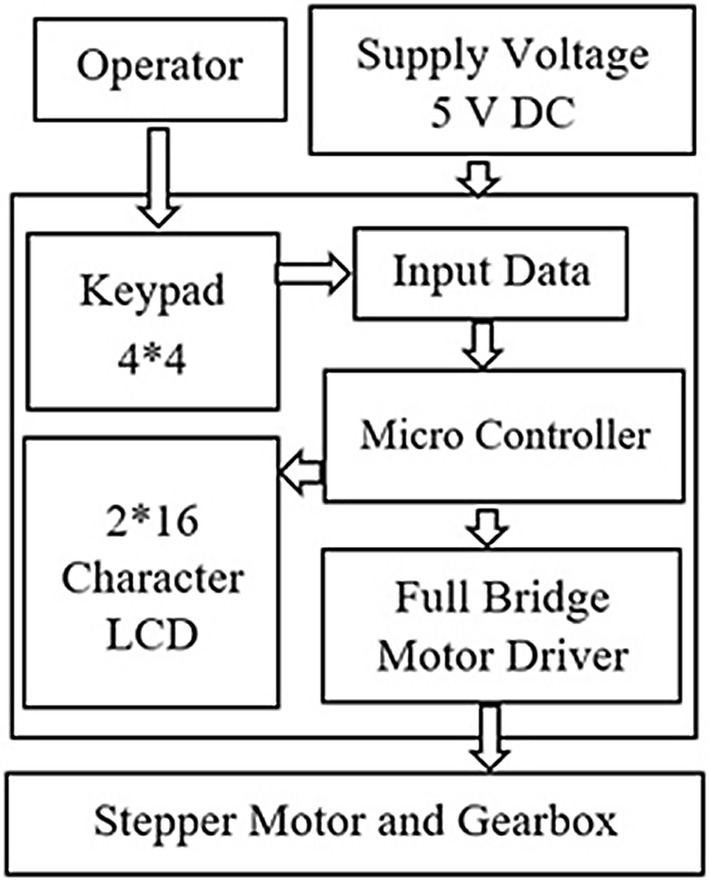
The block diagram of the ACDO controller

Figure 4 shows the block diagram of the ACDO controller, the controller has the capability to control and drive stepper motors with an L298 dual full-bridge driver. The outputs of the controller are connected to the mini stepper motor and gearbox, which is connected to the TM. Every step of the DO process is programmed and controlled in the developed ACDO device by an AVR micro controller with an open loop control system. As DL, DR, and distraction time (DT) are parameters vary with patients’ conditions, the surgeon needs to set these parameters by a removable packed keypad and 2*16 character liquid crystal display panel in a programmed human–machine interface. A programmed ATmega32A 8-bit AVR micro controller is used to get the input data (DL, DR, and DT) from the user and to calculate the distraction parameters (including the steps rate and rhythm), and to save the distraction data (DD) in an AT24C02A serial eeprom with a real-time backup process.

**Fig. 5:**
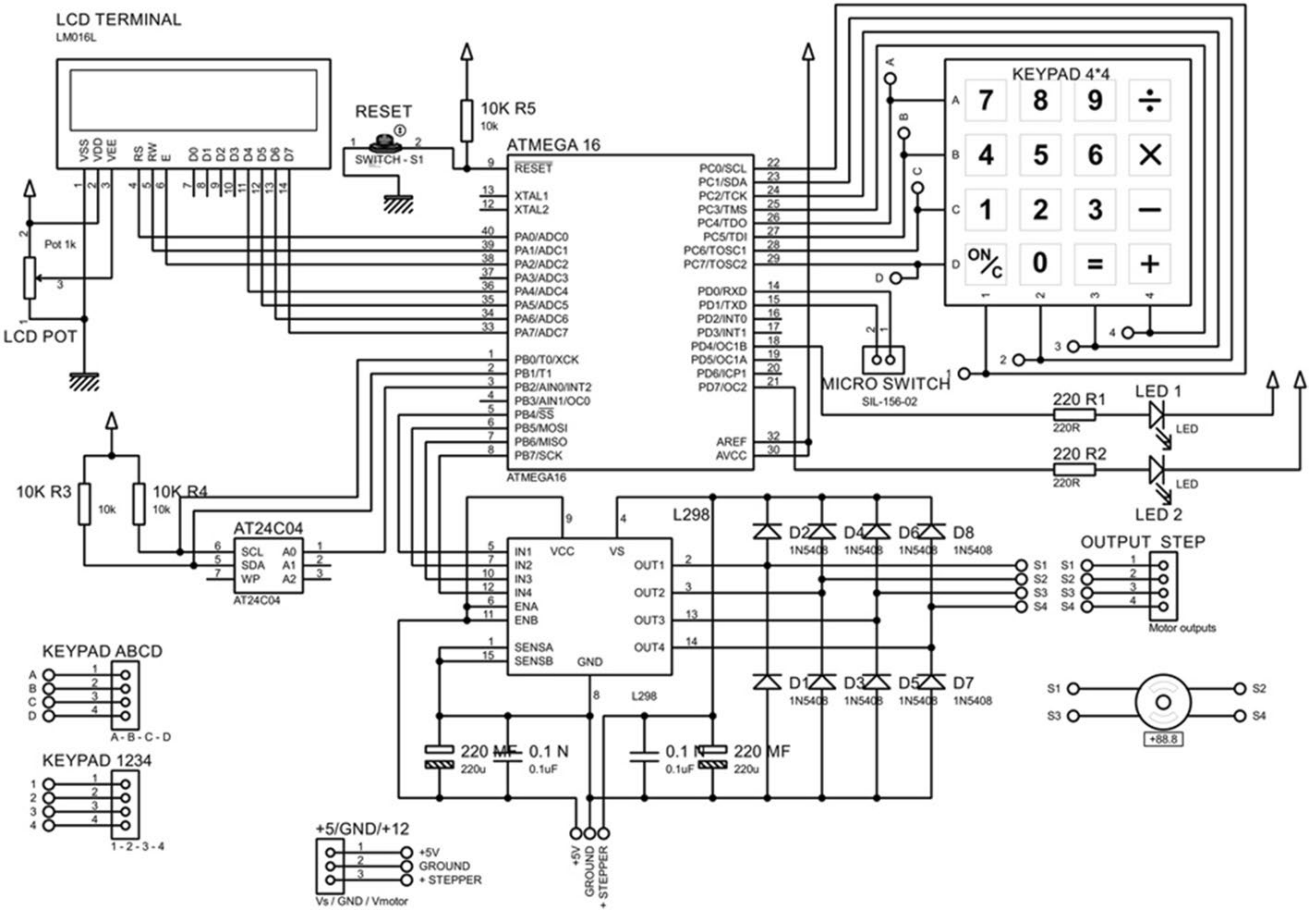
The controller circuit of the ACDO device

In addition, in another subsequent connection, the DL, the DR, and the DT are displayed on the display panel, this feature helps to monitor and edit the distraction parameters whenever required. A 32.768 kHz real-time clock oscillator is also applied with the controller to provide an accurate 8-bit internal timer. Figure 5 shows the designed and implemented controller circuit of the ACDO device.

### Modeling and simulation of the motor

The ACDO controller could drive the stepper motor in three different working states with varied linear and angular movement as shown in Table 2. Micro-step driving method provides improved motion stability and resolution, while increasing the step accuracy and system’s performance compared to full- and half-step driving techniques. It is implemented by partially exciting different phase windings at the same time. Using micro stepping will also improve the movement by eliminating low speed ripple and resonance effects to satisfy the application [55–58]. The mathematical equations of the hybrid stepper motor are given below, which are differential equations of the dynamic model of the motor; (1) and (2) are the electrical equations, and (3) and (4) are mechanical equations [59].

1 $$ \frac{dia}{dt} = \frac{{va + km \cdot \omega \cdot sin\left( {N \cdot \theta } \right) - Ria}}{L} $$

2 $$ \frac{{d{\text{ib}}}}{dt} = \frac{{vb + Km \cdot \omega \cdot cos\left( {N \cdot \theta } \right) - Ria}}{L} $$

3 $$ \frac{d\omega }{dt} = \frac{{Km \cdot ib \cdot cos\left( {N \cdot \theta } \right) - T - Km \cdot ia \cdot sin\left( {N \cdot \theta } \right) - Kv \cdot \omega }}{J} $$

4 $$ \frac{d\theta }{dt} = \omega $$

**Fig. 6:**
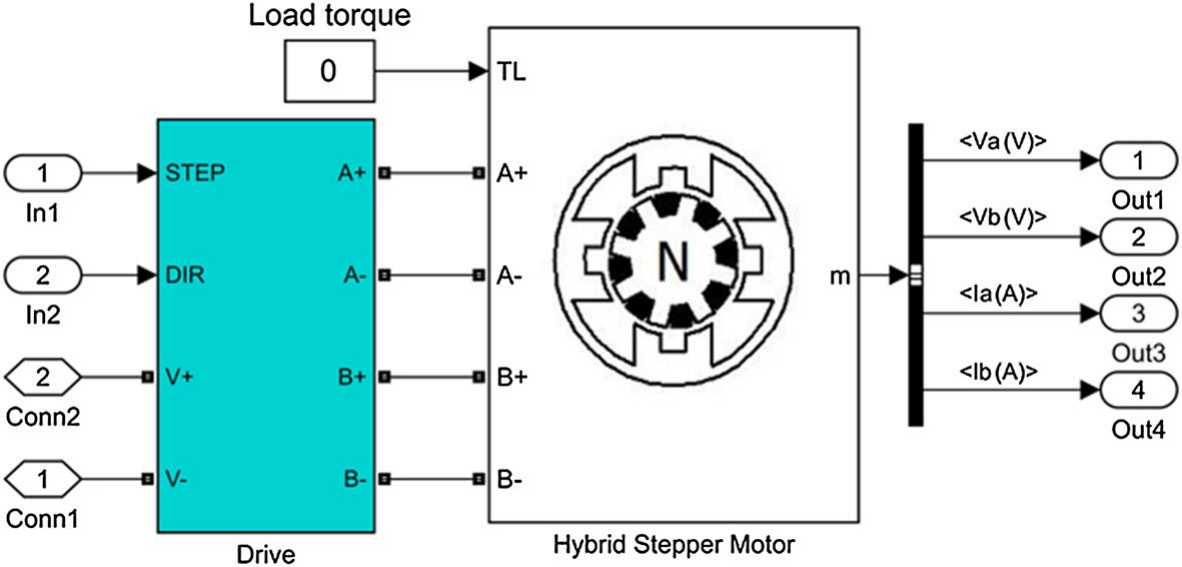
The current subsystem

**Fig. 7:**
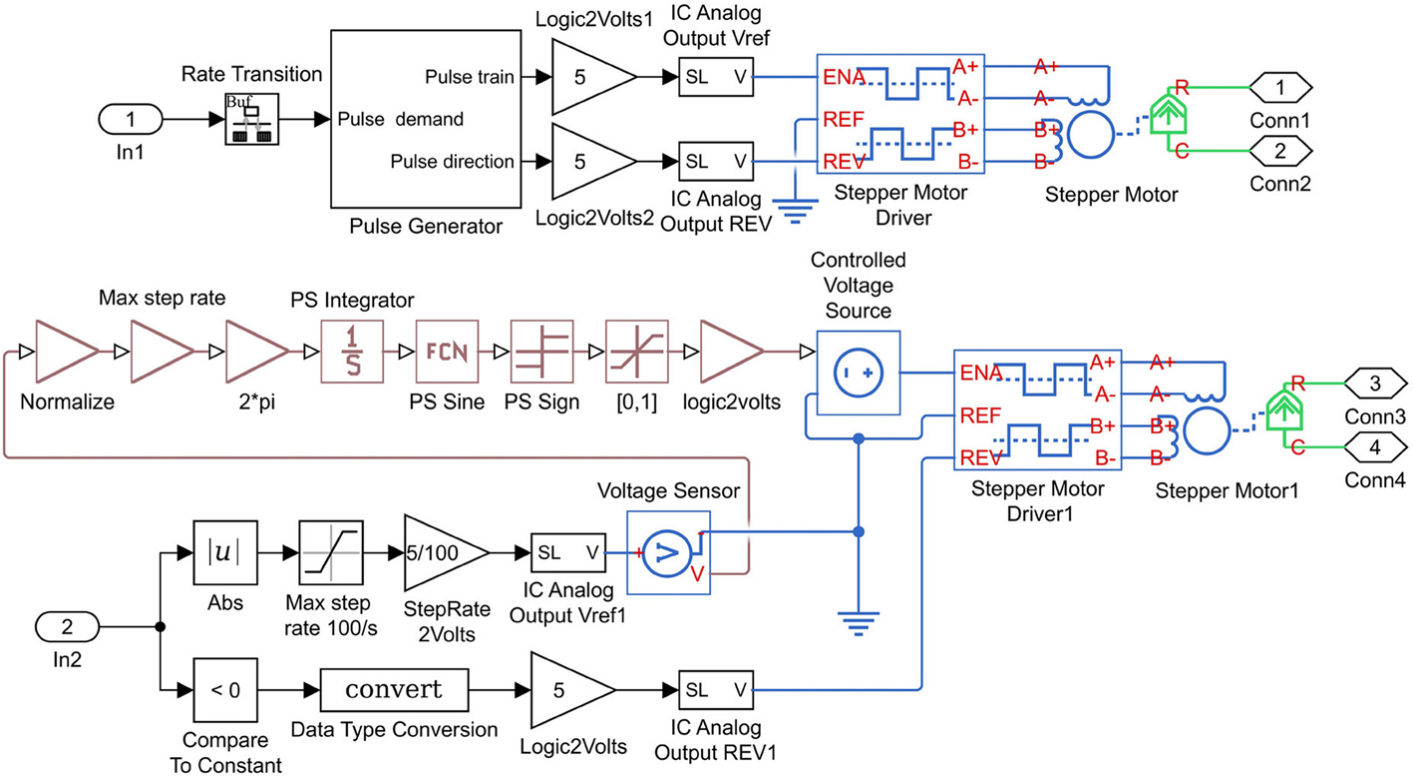
Speed and position subsystems

**Fig. 8:**
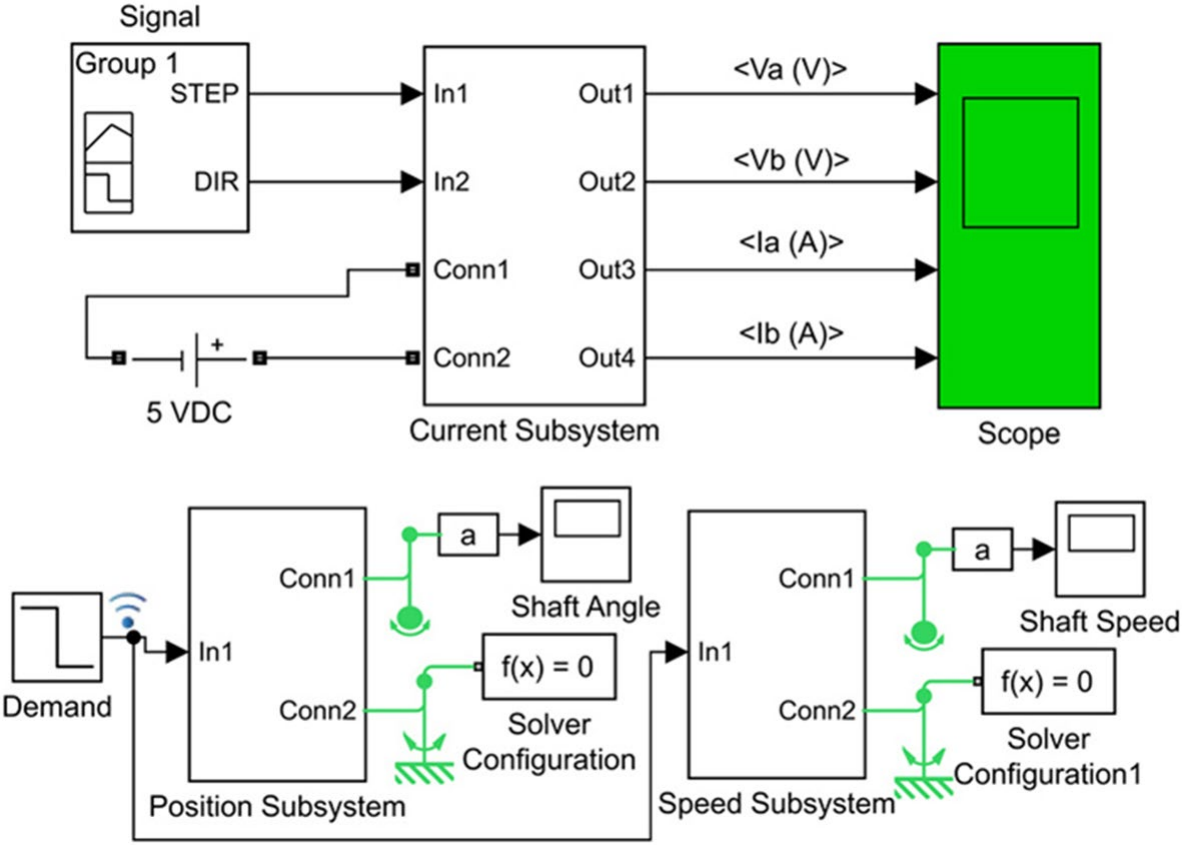
The overall simulation model of stepper motor

In the given equations; ia (the current) and va (the voltage) are the parameters of phase A, ib (the current) and vb (the voltage) are the parameters of phase B, ω is the rotor rotational speed (rad/s), T is the load torque (N m), and ϴ is the rotor angular position (rad). Some modeling factors are neglected in the modeling of the motor, including detent torque, the change in inductance, and magnetic coupling between phases. For evaluating the design and the selected movement technique, the model and the simulation of the stepper motor implemented in MATLAB-SIMULINK. Figure 6 shows the subsystem of the current based on Eqs. (1) and (2). Figure 7 shows the subsystem of speed and position based on Eqs. (3) and (4). The simulated model of the stepper motor and the diagrams are shown in Fig. 8.

### Flexible shielded transition system

**Fig. 9:**
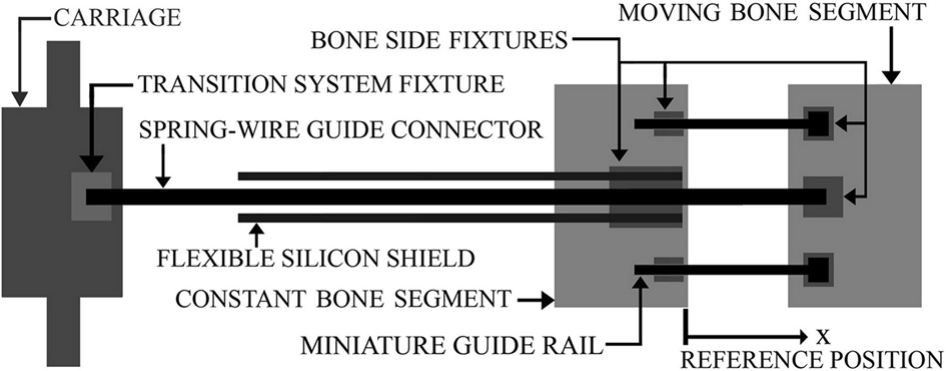
The schematic model of the TS

The TS consists of a flexible miniature single-lumen catheter and a flexible stainless-steel spring-wire guide to transfer the DF to the callus. Figure 9 shows the schematic model of the designed TS in the ACDO device. A mechanical fixture placed on the carriage transfers the linear DF to the spring-wire connector. The generated linear DF pushes the shielded spring-wire connector and the DF totally transfers to the moving BS throughout the flexible single lumen catheter. One side of the spring-wire guide is connected to the mechanical fixture on the TS and the other side is connected to another fixture on the mechanical part of distractor placed on the moving BS. The mechanical part placed on the bone side consists of one 3*3*10 mm stainless-steel solid fixture to fix the end of flexible shield to the constant bone part, one 3*3*3 mm stainless-still solid fixture to fix the end of the spring-wire connector to the moving BS, and two custom-designed 3*3*25 mm stainless-steel miniature guide rails to provide a stable distraction in the desired DV with a maximum travel of 22 mm. Four 1.5-mm holes drilled into the constant bone part and 3 other similar holes drilled into the moving BS. Subsequently, seven bio-compatible self-tapping titanium bone screw, diameter of 2-mm and 6-mm long (TREC, Germany), are used to fix these mechanical components to BS and to provide a linear DV. Each movement command generated by the controller drives the motor in micro stepping drive mode and the carriage moves forward 7.63 nm, consequently, the spring-wire connector pushes and the BS moves 7.63 nm.

### Experimental Setup

**Fig. 10:**
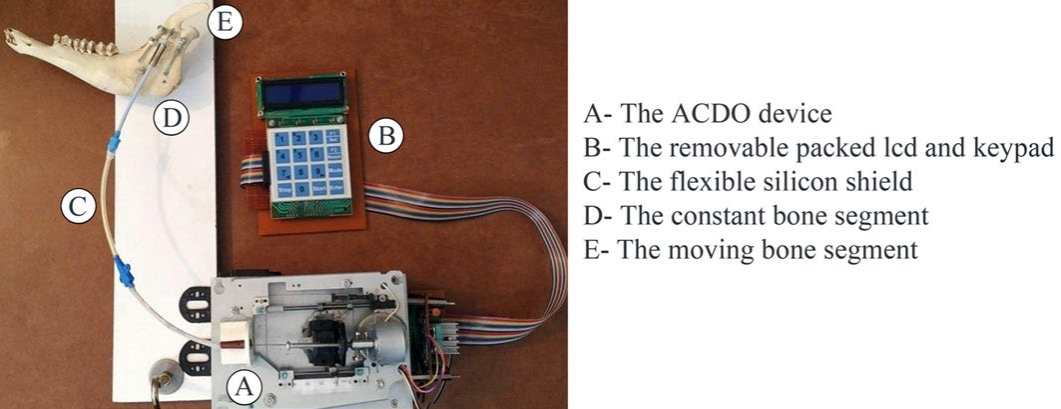
The device connected to the sheep jaw bone

Following the design and development of the ACDO device, experiments have been performed on a sheep jaw bone distraction model. In this experiment the jaw bone of a two-year female sheep is used. The similarity of the sheep jaw bone to human consists of anatomic, macroscopic, and physiologic properties [5, 60]. Based on the literature and specifications of the existing devices it can be deduced that a typical DO treatment for different cranio-maxillofacial areas including; mandible, alveolar bone, mid-face, and cranio-orbit, involves a DL of 10 to 20 mm, a DR of 1 to 3 mm/day, and a DT of 7 to 10 days [15, 25, 33]. To cover all clinical conditions of the treatment, six different tests with various repeat cycle, DT, DL, and DR are carried out with the predetermined factors shown in Table 3. Figure 10 shows the developed device connected to the jaw bone. The DR, the DT and the DL are measured in all experimental tests with an 8-bit digital timer–counter and a Mitutoyo digital caliper 0–300 mm with the precision of 0.01 mm and the resolution of 0.01 mm. These parameters have been considered to calculate the DO procedure results and the error percentage of factors with different input data. Statistical analysis was performed with descriptive tests, and graphical results were generated by using MATLAB software.

**Table 3:** Predetermined factors of the tests

Test	Repeat cycle	DT (h)	DL (mm)	DR (mm/day)
A	10	48	10	5
B	10	96	20	5
C	10	80	10	3
D	5	160	20	3
E	5	240	10	1
F	2	480	20	1

**Fig. 11:**
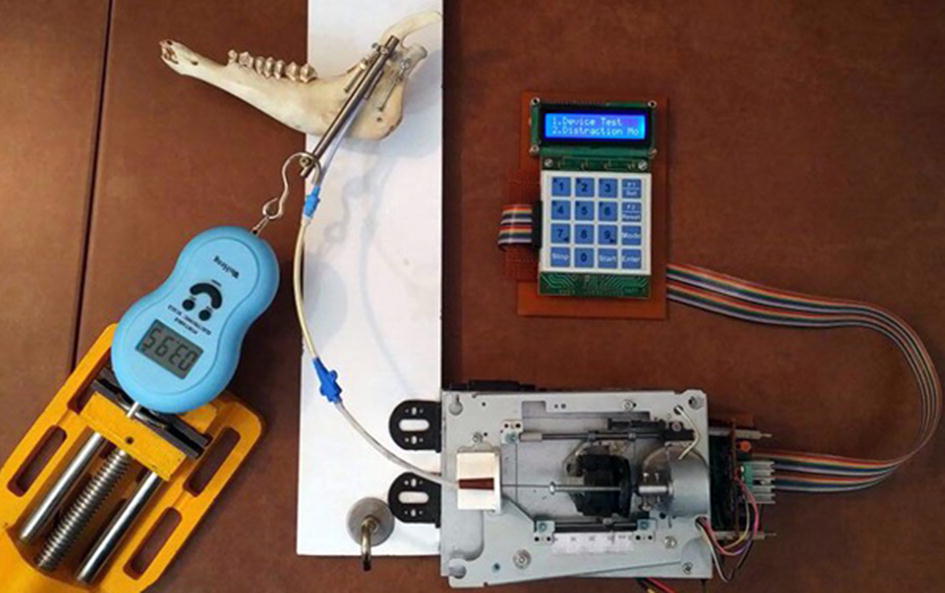
The DF measurement exam

For the DF measurement, a standardized testing environment with approximate temperature of 30 centigrade and atmospheric pressure of 1*10^5^ Pa was used. The maximum generated DF is then measured with a horizontally fixed WeiHeng digital spring scale DP-G004 with the accuracy of 0.1 N. Figure 11 shows the carriage connected to the fixed digital scale for DF measurement.

## Results

**Fig. 12:**
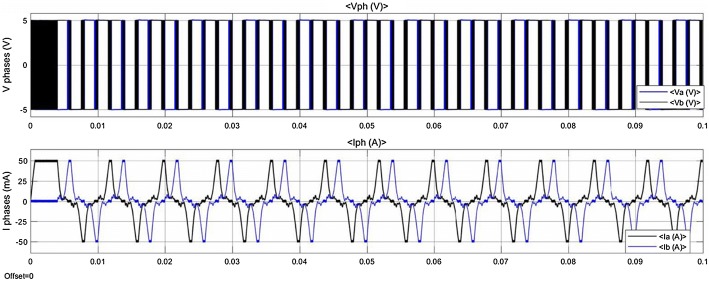
Simulation results of the stepper motor

**Fig. 13:**
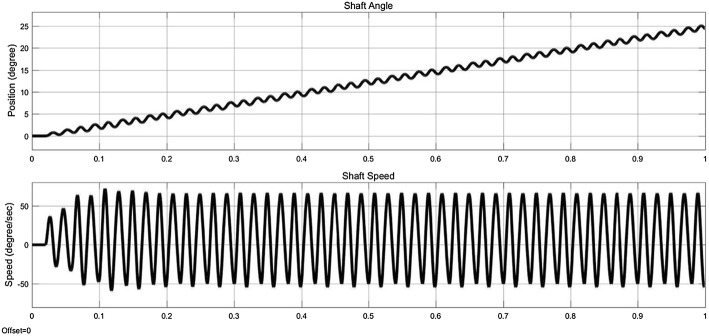
Simulation results of the stepper motor

The controller, drives the motor in the micro stepper mode with an open-loop control method. After all parameters of the selected motor are defined in the designed model, the simulation is run. The detailed waveforms, as shown in Fig. 12, are the outputs of the simulated model. Time for the simulation execution is defined one second. The Ia waveform, shows the electric current in phase a, and the Ib waveform, shows the electric current in phase b. In same way, the Va waveform shows the voltage in phase a, and the Vb waveform shows the voltage in phase b. The rotational speed of the stepper motor and the shaft’s position are the other simulation outputs, as shown in Fig. 13.

In all test conditions, the movement of the moving BS was easily achieved without any failure in the mechanical and electrical part of the device. The recorded movement is accurate and stable. The mean measured distraction length (MMDL) and the mean calculated distraction rate (MCDR) of the tests are summarized in Table 4. The corresponding mean measured distraction length error, the mean calculated distraction rate error, the mean calculated step error, the DR error rate, the DL error rate, and the mean calculated step error rate of tests are summarized in Table 5. Results have shown that all test groups had expected results with the step error rate less than 6%, DL error less than 1%, and the maximum DR error rate of 4%, respectively.

**Table 4:** The mean measured factors of the tests

Test	A	B	C	D	E	F
MMDL (mm)	10.07	20.16	10.09	20.17	10.03	20.05
MCDR (mm/day)	5.03	5.04	3.02	3.02	1.003	1.002

**Table 5:** The mean measured errors of the tests

Test	A	B	C	D	E	F
Mean measured distraction length error (mm)	0.07	0.16	0.09	0.17	0.03	0.05
Mean calculated distraction rate error (mm/day)	0.03	0.04	0.02	0.02	0.003	0.002
Mean calculated step error (nm)	0.05	0.06	0.06	0.06	0.02	0.02
DL error rate (%)	0.7	0.8	0.9	0.85	0.3	0.25
DR error rate (%)	3	4	2	2	0.3	0.2
Mean calculated step error rate (%)	5	6	6	6	2	2

**Fig. 14:**
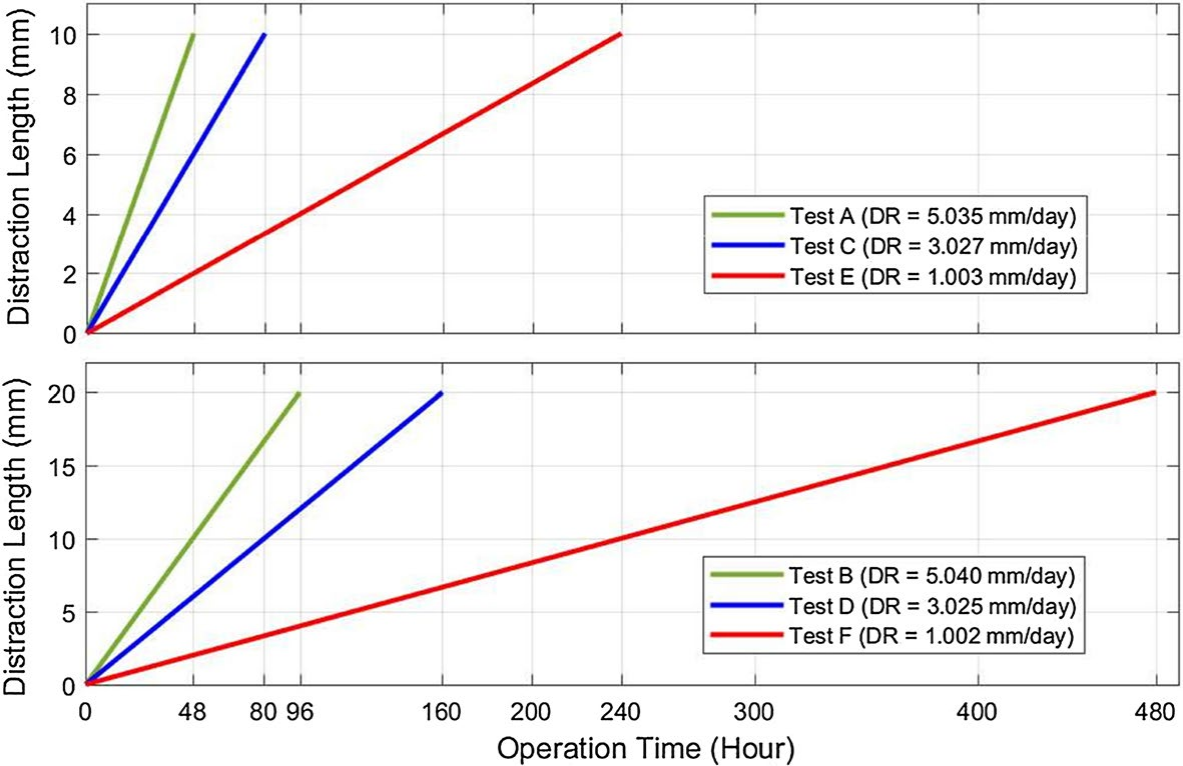
The mean measured DT and the MMDL of the tests

Figure 14 shows the MMDL and the mean measured DT of the test groups. Another experiment was done to measure the continuous DF generated by the device and the result has shown that in all test conditions, the device had generated a DF of 38 N during the distraction.

## Discussion

DO is a recent technique regularly used in MRA, the success of this treatment depends on the rate and the rhythm of distraction, the generated DF, and the DV [13, 24, 26, 36]. Different methods have been used for developing ACDO devices and improving such influencing factors. In spring-mediated continuous distractors, the reduced spring force and the nonlinear DV are major limitations [5, 18, 33]. In motor-based automatic distraction devices, due to the attached gearbox, the size increased and may cause bone fracture, and post-operative infections [12, 26, 43, 60, 61]. The main limitation of hydraulic devices is that the distractor is not able to generate a constant amount of DF and there is a load peak when device executes distraction. In addition, the intra-oral valve and the tube connection have a bigger size and increase the risk for infection and bone fracture [40, 44, 51]. Another general problem is software related issues which causes instability, measurement errors, and restarting the whole process [7, 32, 44]. In general, motor-based systems offer more suitable controllability, distraction accuracy, reliability, actuation power, and biocompatibility compared to other mechanisms [35]. Table 6 shows specifications of the existing motor-based and hydraulic ACDO devices.

**Table 6:** The existing ACDO devices and their specifications

Refs.	Year	Mechanism	Distraction accuracy (μm)	Distraction step error (μm)	Operated distraction rate (mm/day)	Maximum travel (mm)	Distraction force (N)	Total size (mm)
[60]	1999	Motor-based	40	0.5	1	13.6	–	–
[51]	2000	Hydraulic	–	–	1.5	–	30 to 50	–
[40]	2004	Motor-based	1000	20	1	15	70	60
[62]	2005	Hydraulic	–	–	1	16	20	–
[25]	2008	Motor-based	–	80	0.9	10	19	55
[44]	2009	Hydraulic	10	86	1	25	25 to 40	30 to 100
[26]	2009	Syringe pump	–	21,000	0.9	15	–	–
[35]	2010	Motor-based	600	–	–	15	35.6	–
[24]	2011	Motor-based	200	2000	1.4	7	2.84	35
[63]	2011	Motor-based	0.75	30	3	3	–	–
[7]	2013	Hydraulic	–	Average < 500	1.5	12	25 to 40	18
				Average < 1000	3			
				–	4.5			
[5]	2014	Motor-based	300	4	2.4	18	–	–
[32]	2015	Hydraulic	–	–	3	30	25 to 40	–
Proposed device		Motor-based	0.00763	0.00006	1	22	38	25
				0.00006	3			
				0.00002	5			

The minimum DF needed for moving the BS is about 35 N [24, 35, 44, 51, 64–66], in addition, the distractor should allow continuous extension of the BS with a constant DF avoiding high loading peaks and tissue damage [51]. According to Table 6, hydraulic devices are capable to generate an average DF of 25 N with a load peak of 40 N [7, 32, 44, 51], while motor-driven systems are capable to generate a constant amount of DF. Two of motor-based distractors are capable to generate sufficient DF for a DO procedure, however, they are limited in distraction accuracy, DR, and DL [35, 40]. The most accurate distractor in existing devices is a motor-based system; the distraction accuracy of this device is 0.75 Â µm, the step error is 30 µm, and the DR is 3 mm/day [63]. The objective of this study was to design and develop a high-precision ACDO device for bone distraction, and to provide a constant amount of DF for a soft and continuous distraction, while decreasing the size of intra-oral distractor. The proposed device is equipped with an extra-oral MAAC controller capable of controlling the system in different conditions while driving in a linear axis with the maximum position accuracy of 7.63 nm. In addition to enabling high levels of distraction accuracy, the stepper motor in micro-stepping drive mode has provided a much smoother movement, less vibration and noiseless operation; it lowers system complexity and cost. This is due to the stator flux, which is moved in a more-continuous way compared to other drive modes, and causes a precise and smooth control of the rotor stop position [54–57], consequently, a soft continuous distraction for the BS. From the results of the simulation it can be deduced between two phases of the stepper motor, voltage waveforms are 90° displaced, in addition, current waveforms of the phases are alike to sine and cosine waveforms with 90° displacement. Simulation results show that the designed control system and the driving method used in this device, work well in different conditions, and agree with the theoretical equations. Furthermore, experimental tests have been carried out by varying the DR from 1 to 5 mm/day, the DL from 10 to 20 mm, and the DT from 48 to 480 h. Results have shown that the device has an accurate movement with the DL error rate less than 1% and the DR error rate less than 4% in all experimental test phases with great repeatability, respectively. The measured output force including a preload in the axial direction showed that in all test conditions as shown in Table 3, the pushing DF during the distraction has a value of 38 N. Therefore, the device has the capability to sufficiently provide a constant DF in different DO conditions, respectively. In addition to improved distraction accuracy and smoother DF, the size of the mechanical part placed in the oral cavity is decreased to 25 mm. The device is equipped with a simple and user-friendly human–machine interface with liquid crystal display and keypad for programming and debugging. This feature will allow the user to set various DO working factors, check, or modify the working parameters during the DO procedure. The serial eeprom connected to the controller provides a real-time backup system, and the controller can read the DD at any moment. In the case of unwanted error or system failure, the device is capable of reading and recovering the DD and continuing the distraction procedure without any movement errors.

There were some limitations in this study as well. The ex vivo model test is limited and no clinical prospect can be directly deduced from it. The software simulation was limited to the motor simulation only. In the motor simulation, some of influencing factors including detent torque, the change in inductance, and magnetic coupling between phases were neglected. The experimental tests were limited by use of a single Jaw bone model. The device was fabricated all in house and it was limited in selecting materials and fabricating complicated parts. However, the prototype served well in demonstrating the design concept and functionality for automatic continuous DO procedure.

## Conclusion and future works

A newly designed ACDO device with using mini motor and gear box, miniature TM, and TS is developed for the MRA which has met all the necessary mechanical and medical functions. The experimental test results have validated its stability, reliability, and movement accuracy. The device has less than 1% positioning error with sufficient DF, while generating continuous force. The DR can be adjusted accordingly to reduce the activation phase and the DT in the DO process. Usage of a simple and ongoing control and monitoring interface makes the device easy to use. The design of the on-line DD backup plan makes the system stable and reliable for unwanted failures, and there will be no need for surgery for failed software and controller. The miniature flexible TS and the small size of the mechanical part placed on the callus, has increased the potential of the device for different cranio-maxillofacial areas including; mandible, alveolar bone, mid-face and cranio-orbit. This device is a suitable distractor for animal studies; in the future, it will be tested in the human MRA as an enhanced continuous DO solution. Additional improvements can be made on several areas to maximize its future potential and success, such as on the DV, reducing the size of the device, and making a wireless communication system for the packed display and keypad panel to enable an ongoing monitoring system showing the working DD. Developing a rechargeable high-power battery system with a design of an electronic gauge and a low-battery alarm system could make this device more suitable for MRA in human.

## Abbreviations

DO: distraction osteogenesis
MRA: maxillofacial reconstruction applications
ACDO: automatic continuous distraction osteogenesis
DF: distraction force
DR: distraction rate
DV: distraction vector
BS: bone segment
TM: translation mechanism
TS: transition system
DL: distraction length
DD: distraction data
DT: distraction time
MMDL: mean measured distraction length
MCDR: mean calculated distraction rate
